# Evidence for the recent origin of a bacterial protein-coding, overlapping orphan gene by evolutionary overprinting

**DOI:** 10.1186/s12862-015-0558-z

**Published:** 2015-12-18

**Authors:** Lea Fellner, Svenja Simon, Christian Scherling, Michael Witting, Steffen Schober, Christine Polte, Philippe Schmitt-Kopplin, Daniel A. Keim, Siegfried Scherer, Klaus Neuhaus

**Affiliations:** Lehrstuhl für Mikrobielle Ökologie, Wissenschaftszentrum Weihenstephan, Technische Universität München, Weihenstephaner Berg 3, 85350 Freising, Germany; Lehrstuhl für Datenanalyse und Visualisierung, Fachbereich Informatik und Informationswissenschaft, Universität Konstanz, Box 78, 78457 Constance, Germany; Lehrstuhl für Ernährungsphysiologie, Wissenschaftszentrum Weihenstephan, Technische Universität München, Gregor-Mendel-Straße 2, D-85354 Freising, Germany; Research Unit Analytical BioGeoChemistry, Deutsches Forschungszentrum für Gesundheit und Umwelt GmbH, Helmholtz Zentrum München, Ingolstädter Landstraße 1, 85754 Neuherberg, Germany; Institute of Communications Engineering, Universität Ulm, Albert-Einstein-Allee 43, 89081 Ulm, Germany; Present address: Blue Yonder GmbH, Ohiostraße 8, Karlsruhe, Germany; Present address: Institut für Biochemie und Molekularbiologie, Universität Hamburg, Martin-Luther-King Platz 6, 20146 Hamburg, Germany

**Keywords:** Overprinting, Overlapping gene, *de novo* evolution, Coding reserve, Orphan, EHEC, *nog1*/*citC*

## Abstract

**Background:**

Gene duplication is believed to be the classical way to form novel genes, but overprinting may be an important alternative. Overprinting allows entirely novel proteins to evolve *de novo*, i.e., formerly non-coding open reading frames within functional genes become expressed. Only three cases have been described for *Escherichia coli.* Here, a fourth example is presented.

**Results:**

RNA sequencing revealed an open reading frame weakly transcribed in cow dung, coding for 101 residues and embedded completely in the −2 reading frame of *citC* in enterohemorrhagic *E. coli*. This gene is designated novel overlapping gene, *nog1*. The promoter region fused to *gfp* exhibits specific activities and 5’ rapid amplification of cDNA ends indicated the transcriptional start 40-bp upstream of the start codon. *nog1* was strand-specifically arrested in translation by a nonsense mutation silent in *citC*. This Nog1-mutant showed a phenotype in competitive growth against wild type in the presence of MgCl_2_. Small differences in metabolite concentrations were also found. Bioinformatic analyses propose Nog1 to be inner membrane-bound and to possess at least one membrane-spanning domain. A phylogenetic analysis suggests that the orphan gene *nog1* arose by overprinting after *Escherichia/Shigella* separated from the other γ-proteobacteria.

**Conclusions:**

Since *nog1* is of recent origin, non-essential, short, weakly expressed and only marginally involved in *E. coli*’s central metabolism, we propose that this gene is in an initial stage of evolution. While we present specific experimental evidence for the existence of a fourth overlapping gene in enterohemorrhagic *E. coli*, we believe that this may be an initial finding only and overlapping genes in bacteria may be more common than is currently assumed by microbiologists.

**Electronic supplementary material:**

The online version of this article (doi:10.1186/s12862-015-0558-z) contains supplementary material, which is available to authorized users.

## Background

A widely established model to explain the evolutionary origin of novel genes is gene duplication [1–4]. However, recent phylogenetic evidence suggests that *de-novo* formation might be an alternative, important source for the *de novo* origin of orphan genes [5]. This is corroborated by findings that long non-coding RNA may serve as a novelty pool and that ribosomes indeed translate novel ORFs [6, 7]. It is hypothesized that this mechanism might produce novel domains or folds, which are added to existing genes or assembled to new genes [8, 9].

In eukaryotes, large parts of the genome do not harbor protein-coding genes, potentially providing DNA raw material for novel genes [10, 11]. In contrast, prokaryotic genomes are densely packed with genes and inter-genic space is quite limited. Therefore, as early as 1977, Grassé proposed a mechanism for the evolution of novel genes termed “overprinting” [12], which some years later was substantiated by Ohno [13]. According to this hypothesis, a previously non-coding sequence, overlapping an existing gene in an alternate reading frame, is transformed into a coding sequence by the creation of a new promoter next to a suitable ribosome binding site and a start codon. Alternatively, a gene may elongate through the emergence of an alternative start codon further upstream or the loss of its original stop codon, leading to an overlap with an adjacent gene. This mechanism of overprinting is an option to solve the *de-novo* evolution problem for prokaryotes.

Trivial overlaps of only a few base pairs are found in about 30 % of the bacterial genes [14, 15]. The likely benefit is a translational coupling of both genes, since the stop codon of the upstream located gene overlaps with the start codon of the downstream gene [16]. In non-trivially overlapping genes the protein coding regions are embedded completely or substantially in the annotated “mother gene”, which by definition occupies reading frame +1, and are encoded by one of the five alternate reading frames. Non-trivially overlapping genes are generally assumed to be very rare. This assumption is due to a severe information content constraint since single mutations often affect the protein function of both overlapping genes. Thus, such an arrangement is believed to be less likely to be beneficial for the organism carrying the overlapping gene pair [14].

The majority of non-trivial overlapping genes have been described in viruses [17–20] and their emergence was attributed to a hypothetical selection pressure acting on the size of the viral genome, exerted by spatial limitations of the capsid [21]. In sharp contrast, in prokaryotes only very few overlapping gene pairs are known. In the extremely well-researched *Escherichia coli*, as far as we know, only three overlapping gene pairs have been described: *htgA*/*yaaW* [22, 23], *yghW*/*morA* [24], and *tnpA*/*astA* [25, 26].

Here we report on direct experimental evidence indicating the presence of a novel overlapping gene pair in enterohemorrhagic *E. coli* O157:H7 str. EDL933 (EHEC) which was found during the investigation of the transcriptomic response of EHEC to a number of environmental conditions [27]. The novel overlapping gene *nog1* is completely embedded −2 antisense in its mother reading frame *citC* which is part of the operon *citCDEFXG.* This operon is responsible for citrate fermentation. *citC* is induced anaerobically and encodes the citrate lyase ligase which activates the γ-subunit of the citrate lyase [28]. In addition to a functional analysis of *nog1*, we provide evidence that this overlapping gene may be restricted to the *Escherichia*/*Shigella*/*Salmonella* clade and probably arose recently by genetic overprinting.

## Methods

Bacterial strains and plasmids are listed in Additional file 1: Table S1.

### Construction of *gfp* fusions and fluorescence measurement

The region 365 bp and 361 bp upstream of the start codons of *citC* and *nog1* respectively was amplified from genomic EHEC-DNA (NC_002655, [29]) using primer GGC G*GT CGA C*cg gtg cct ttt aac acc aga tc (Z0762 + 667R-Sall) and ACA *GAA TTC* gaa ctg ata aac ctc gcc tat g (Z0762+325F-EcoRI) and the primers GGC G*GT CGA C*aa aga tac gca gcg gaa atg c (Z0762-362F-SalI) and ACA *GAA TTC* tgg gag aaa ggg ggg tga tcg a (Z0762-3R-EcoRI) respectively. The PCR products were digested with the appropriate enzymes (cut sites italic above) according to the manufacturer (NEB) and ligated in pProbe-NT [30] using T4-ligase (NEB). EHEC was transformed with the plasmids and was grown under shaking at 37 °C in the dark in LB medium [31] supplemented with 25 μg/ml kanamycin. For aerobic growth, 1:100 diluted overnight cultures were used to inoculate 10 ml 1:2 diluted LB with 25 μg/ml kanamycin and cells were grown at 37 °C for 4 h until the culture reached OD_600nm_ = 1 in plain LB medium. Induction of the promoter fusion was tested using 100 mM 1,2-propanediol, 20 mM CsCl, 1.25 mM CuCl_2_, 25 mM cycloheximide, 1 mM dithiothreitol, 78 μM erythromycin, 200 mM formamide, 4 mM HCOOH, 2 mM glutamine, 200 mM MgCl_2_, 320 μM menadione, 400 mM NaCl, 10 mM 1-methylimidazole, 10 mM propanedioic acid, 62.5 mM salicin, 16 mM Na_3_VO_4_, and 10 mM Na_2_B_4_O_7_.

For anaerobic growth, bacteria were grown in 15 ml medium as above in tightly closed 15-ml falcons. Anaerobiosis was tested using resazurin in separate tubes. All cultures, including an empty-vector control, were grown for approximately 7 h to OD_600nm_ = 0.3 in the dark. To allow GFP to mature to fluorescence, anaerobically grown cultures were aerated for 15 min by shaking in larger bottles. Bacteria were washed once with 1 ml PBS (6600 × *g*, 2 min) and diluted to OD_600nm_ = 0.6. Two-hundred μl of this suspension was measured for fluorescence using a black microtiter plate and a plate reader (Wallac Victor^3^, Perkin Elmer Life Science, excitation 485 nm, emission 535 nm, measuring time 1 s). The mean value of four replicate wells was calculated and the experiment was repeated three times.

### Determination of the transcription start site by 5’-RACE

5’-RACE was performed using the “5’RACE System for Rapid Amplification of cDNA Ends Version 2.0” (Invitrogen) according to the manufacturer. Wild type strain was grown in 1:2 diluted LB medium with the addition of 320 μM menadione to OD_600nm_ = 0.5. RNA was isolated with Trizol. For 5’-RACE, the primers CAA CAT GCA CCT TCA GGA T (Z0762+59R) and TGG CGG AAA TCG CCC AAT TCC TGC AT (Z0762+140R) were used. After gel electrophoresis, the strongest band was cleaned (Invisorb® Fragment CleanUp, STRATEC, Berlin) and used as a template for subsequent amplification and sequencing (LGC Genomics, Berlin) using the nested primer GAG CGT TGA CAC CAC AGT CGA AGT AT (Z0762+177R).

### Overexpression of Nog1 C-terminal fused with GFP

The open reading frame of *nog1* was amplified via PCR using primers Z0762+323R-SphI (TT*G CAT GC*C **GTG** GCT AAT GTC AGC GCC AG) and Z0762+20F-KpnI (C *CGG TAC C*CG GTT TGC AAC ATT GAA CAA CA). The amplicon was cloned in the *Sph*I and *Kpn*I restriction sides of pEGFP (CLONTECH laboratories). The plasmid was sequenced for verification. Ten ml LB with 120 μg/ml ampicillin was inoculated with 100 μl overnight culture of TOP10 transformed with the empty vector and the vector containing the open reading frame of *nog1*. Bacteria were grown to OD_600nm_ = 0.3 – 0.5 at 37 °C shaking. 1 mM IPTG was added and bacteria were grown for two more hours. Bacterial culture was washed with 2 ml PBS and subsequently suspended in PBS with OD_600nm_ = 1. Two-hundred μl bacterial suspension was transferred in one well of a black microtiter plate and measured using a plate reader (Wallac Victor^3^, Perkin Elmer Life Science, excitation 485 nm, emission 535 nm, measuring time 1 s). The mean value of four replicate wells was calculated and the experiment was repeated three times.

### Overexpression of Nog1 and Western blot

The open reading frame of *nog1* and its upstream region was amplified via PCR using primer GAT C*CC ATG G*CG GTG CCT TTT AAC ACC AGA TC (Z0762+667R-NcoI) and GAG C*GA ATT C*G TTT GCA ACA TTG AAC AAC ATT (Z0762+21F-EcoRI). The amplicon was cloned in the *Nco*I and *Eco*RI restriction sites of pBAD Myc-His C (Invitrogen) resulting in pBAD-*nog1::*Myc-His. The plasmid was sequenced for verification and EHEC were transformed with pBAD-*nog1::*Myc-His or with pBAD Myc-His C as control. One-and-a-half L of 1:2 diluted LB medium supplemented with 120 μg/ml ampicillin and 320 μM menadione were inoculated 1:250 with an overnight culture of the transformants. Bacteria were grown in six 1-L bottles (250 ml medium each), shaking at 37 °C to OD_600nm_ = 0.5. Bacteria were pelleted (10 min, 3500 × *g*) and resuspended in 15 ml lysis buffer included in the QIAexpress® Ni-NTA Fast Start kit (Qiagen). The cells were sonicated six times for 15 s (interval time 0.5 s at 25 % total power, ultrasonic converter tip UW 2200 powered by HD 2200, Bandelin electronics, Berlin) and proteins were purified under native conditions according to the manufacturer’s protocol. From the empty control, a mock sample was ‘purified’. Proteins were precipitated with acetone and Laemmli-buffer was added. The sample was heated for 5 min at 95 °C and loaded on a 15 % SDS-gel. One well was loaded with 5 μl of the PageRuler Prestained Protein Ladder (Fermentas) for size determination. After SDS-PAGE, the proteins were transferred to a PVDF membrane (Amersham/Millipore). Transfer to the membrane was carried out in blotting buffer (50 mM Tris, 39 mM glycine, 0.039 % SDS in 20 % methanol) for 10 min at 150 mA. Next, the membrane was blocked for 1 h in TBS-T (10 mM Tris, 150 mM NaCl, pH8, 0.1 % Tween 20) supplemented with 1 % BSA at room temperature. The membrane was then incubated with 5 ng/μl BD Pharmingen mouse anti-human c-myc-antibody (BD Biosciences) in TBS-T plus 1 % BSA. After washing twice in TBS-T for 10 min, the membrane was incubated for 1 h at room temperature with the second antibody (alkaline phosphatase (AP) anti-mouse chimera, 6 ng/μl) in TBS-T plus 1 % BSA. The membrane was washed twice for 10 min with TBS-T and equilibrated in AP-buffer (100 mM Tris, 150 mM NaCl, 5 mM MgCl_2_, pH 9.5). For the final detection of the fusion proteins, the membrane was incubated with 0.7 mg/ml BCIP and 0.07 mg/ml NBT solution in AP buffer.

### Construction of translationally arrested mutants

Chromosomal DNA was modified for the Δ*nog1* and Δ*citC* mutants using plasmid pMRS101 [32]. For Δ*nog1*, two fragments of this gene were amplified using primers CAT TTT CAT G**A**A GGA ATT GGG (Z0762+152RmutS) and at*a cta gt*A TTT CAC GCC GAA ATA CTC C (Z0762-59F-SpeI) and the primer CCC AAT TCC T**T**C ATG AAA ATG (Z0762+152FmutS) and gc*g ggc cc*A ACA GCG CCT CGT ATT CGG T (Z0762+382R-ApaI). Mutated bases in the primers and the added restriction sites are marked bold and italic respectively. The two *nog1*-fragments are located up- and downstream of the desired mutation and overlap in this area. The fragments were used in a third PCR using primer Z0762-59F-SpeI and Z0762+382R-ApaI to recreate a complete fragment with the mutation. This was conducted accordingly for Δ*citC* using the primer pairs TGT TAT CGA T**C**T **T**CA ACG AAT GT (Z0762+38RmutA) and at*a cta gt*T AAA TCA ATT AAA TCA CTT A (Z0762-171F-SpeI), and primer pair ACA TTC GTT G**A**A **G**AT CGA TAA CA (Z0762+38FmutA) and gc*g ggc cc*G ATT CAC TGA TAG CAA CGC A (Z0762+268R-ApaI). The final PCR products were cloned in the *Spe*I and *Apa*I sites of pMRS101, and transformants grown in LB medium plus 100 μg/ml ampicillin. The high-copy number ori was removed by restriction with *Not*I and subsequent self-ligation. The remaining plasmid contains a second low-copy pir-dependent ori. This plasmid was introduced into *E. coli* CC118λpir via electroporation, cells were grown in LB medium plus 30 μg/ml streptomycin, and the plasmid sequence was verified by sequencing using either primer CTT ATC GAT GAT AAG CTG TC (pMRS101+458R) or TCA ATC ATG CGA AAC GAT CC (pMRS101+184F). Plasmids were transferred to *E. coli* SM10λpir, which enables conjugation, and integrated in EHEC Nal^R^ via plate mating: Each 500 μl overnight culture was mixed, plated on LB agar, and incubated for 24 h at 30 °C. Cells were resuspended in LB and plated on LB-plates containing 30 μg/ml streptomycin and 20 μg/ml nalidic acid. The correct insertion of the suicide plasmid was confirmed using primers pMRS101+184 F and CTT GCG GGT TGT CCC GAG CC (Z0762-272F) for Δ*nog1* or Δ*citC* from genomic DNA of the trans-conjugants. To facilitate a second cross-over, cultures were grown in plain LB to OD_600nm_ = 0.8 and counter-selected on sucrose agar (modified LB-agar without NaCl, supplemented with 10 % sucrose). A PCR fragment using primers TTC AGT CGC GTG GCG CTG TT (Z0762+460R) and CTT GCG GGT TGT CCC GAG CC (Z0762-272F) obtained from the chromosome was sequenced to identify the desired strand-specific mutants of either *nog1* or *citC.*

### Growth curves of individual strains

Overnight cultures of EHEC wild type, Δ*citC* and Δ*nog1* were used to inoculate 100 ml 0.5 × LB medium supplemented with 200 mM MgCl_2_ in 250-ml Schott bottles at an OD_600nm_ = 0.03. Bacteria were grown at 37 °C under shaking (150 rpm) and the OD_600nm_ was recorded at given time points.

### Competitive growth assays

Overnight cultures in LB were adjusted to OD_600nm_ = 1 and the two test strains were mixed in equal numbers. Control samples were taken immediately (t = 0). Half-strength LB medium (supplemented as indicated, e.g. 150 mM MgCl_2,_ or the substances used before for the promoter induction) was inoculated 1:30,000 using the bacterial mixture. Bacteria were grown under shaking at 150 rpm for 18 h at 37 °C, harvested by centrifugation (3 min, 16,000 × g) and boiled for 5 min at 95 °C to release the DNA. Each sample was used as template for PCR with a locus specific primer pair GTT TGC AAC ATT GAA CAA CAT TCG (Z0762+21F), and GAC TGT GGT GTC AAC GCT CAA ATC (Z0762+172R), for Δ*nog1* or Δ*citC* versus wild type. PCR products were purified and sequenced (AGOWA, Berlin) using primer Z0762+21F for Δ*nog1* and primer Z0762+172R for Δ*citC*. For competitive growth experiments between Δ*nog1* and Δ*citC,* PCR products were generated using primer TTC AGT CGC GTG GCG CTG TT (Z0762+460R) and CTT GCG GGT TGT CCC GAG CC (Z0762-272F). The PCR product was sequenced using primer Z0762+460R. After sequencing, the ratios between peak heights from the Sanger sequencing of the alleles were determined and expressed as a percentage of each strain. This experiment was repeated at least three times.

To complement the translational arrested mutant Δ*nog1*, *nog1* was cloned on the arabinose-inducible plasmid pBAD Myc-His C (Invitrogen) using a PCR-product generated with the following primers gat c*cc atg g*ca gtg gct aat gtc agc gcc ag (Z0762+304R-NcoI) and gag cg*a att c*tc agt ttg caa cat tga aca ac (Z0762+18F-EcoRI). The amplicon was cut with *Nco*I and *Eco*RI and ligated into the plasmid using T4-ligase. The plasmid sequence was verified by sequencing. The translational arrested mutant was transformed either with an empty plasmid (control) or with the overexpression plasmid pBAD-*nog1*. A wild type transformed with the empty vector was used as control. These control bacteria and complemented mutants of *nog1* were grown in competitive assays as described above in medium supplemented with 120 μg/ml ampicillin (for plasmid selection) and 0.2 % arabinose (for *nog1* induction). The percentage of “complemented mutants” versus “control bacteria” was determined as described above after 18 h of competitive growth.

### Metabolomics

For a full description of the metabolome measurements see Additional file 2: File S1. In short, the following strategies have been applied.

#### Metabolomes by GC-MS

The methanol-soluble part of the metabolome was determined from six biologically independent cultures of mutant and wild-type, respectively. Briefly, flash frozen bacteria were extracted derivatized for metabolite analysis via GC-MS in a two-step procedure as described [33]. For metabolite profiling a HP Agilent 7890 gas chromatograph was used to perform GC analysis and coupled to an Agilent 5975 Quadrupole mass spectrometer (Agilent Technologies, Böblingen) for mass determination. The software MetaboliteDetector (version 2.06) was used for processing. Differences in the metabolome between wild type and the mutants were tested for significance using Student’s test (T-test; p ≤ 0.05).

#### Metabolomes by ICR-FT/MS

Sample preparation was carried out as above for three biological replicates of each wild type, Δ*nog1* and Δ*citC* mutants. Metabolite profiling was conducted using Ion cyclotron resonance Fourier transform Mass spectrometry (ICR-FT/MS) on a Bruker solariX equipped with a 12 T magnet (Bruker Daltonics, Bremen). Putative metabolites were annotated using the MassTRIX webserver [34]. Statistical analysis was carried out in MS Excel 2010 and Genedata Expressionist for MS 7.6 (Genedata, Martinsried) using Welch’s T test (*p* ≤ 0.05) [35, 36].

### Bioinformatics methods

σ^70^ Promoters were searched for using BProm (Softberry Inc., New York), and terminators using WebGeSTer DB [37]. Sequences from other bacteria were searched with blastp or tblastn (NCBI, http://blast.ncbi.nlm.nih.gov/Blast.cgi, default parameters). All methods mentioned in the following were used with preset values. Domain search was conducted using CDD [38] with an e value cut-off of 10^−2^. The distribution of charged amino acids in Nog1 was analyzed manually. Prediction of disordered regions was conducted using GlobPlot [39] and Meta-Disorder [40] The authors of GlobPlot claim for a given prediction a specificity of 88 % and a sensitivity of 28 % [39]. For Meta-Disorder, the preset reliability index suggests to find about 52 % of the disordered residues and 68 % of those would be correct [40]. Low complexity regions were discovered using CDD [38] which implements SDUST with preset values [41]. The transmembrane and overall topology was predicted using hmmtop [42], TMHMM [43], PHDhtm [44], and BCL::Jufo9D [45]. The program hmmtop correctly predicts 89 % of the membrane spanning regions and 71 % of the correct topology [42]. TMHMM correctly predicts 97-98 % of the transmembrane helices. The specificity and sensitivity of this program is greater than 99 % if no signal peptide is present [43]. PHDhtm predicts the topology for helical transmembrane proteins at 86 % accuracy [44]. Finally, BCL::Jufo9D has a prediction accuracies of 73.2 % the secondary structure prediction, and 94.8 % for the transmembrane span prediction [45]. Beta-turns were predicted using NetTurnP [46]. Sensitivity is 76 % and specificity 79 %, respectively, in distinguishing turns from not-turns [46]. The protein secondary structure was established using SOPMA [47], PSIPRED [48], Pred2ary [49], and GOR IV [50] via the web server NPS@ [51]; PROFseq [52] via the PredictProtein server [53], BCL::Jufo9D [45], or using Porter [54]. SOPMA correctly predicts 69.5 % of amino acids for a three-state description (α-helix, β-sheet and coil; Q_3_) of the secondary structure [47]. The current PSIPRED 3.2 achieves an average Q_3_ score of 81.6 % [55]. For Pred2ary, the secondary structure prediction accuracy was given as 63 % [49], and GOR IV has a mean accuracy of 64.4 % for Q_3_ [50]. PROFseq has an overall accuracy of 71.6 % and a sustained Q_3_ of 88 % for 40 % of the residues [56]. BCL::Jufo9D has prediction accuracies of 70.3 % for nine possible states, and 73.2 % for the Q_3_ secondary structure prediction [45]. Finally, Porter's accuracy exceeds 79 % [54]. Protein binding sites were predicted using ISIS, which has total two-state accuracy of 68 % [57]. Protein statistics were calculated using ProtParam [58], signal protein sequences were searched for using SignalP [59], and the protein localization was predicted using LocTree3. This program reaches a six-state accuracy (6 localizations) of 89 ± 4 % for bacteria [60].

### Tree construction

The evolutionary tree is based on a concatemer of the conserved genes 16S rRNA, *atpD*, *adk*, *gyrB*, *purA* and *recA* and is independent of *citC/nog1*. The gene sequences were obtained from the genome of each organism (for sequences and accession numbers, see Additional file 3: Table S2). All the above-mentioned genes of each organism were concatenated and aligned using muscle in Mega6.06 [61] with default parameters. The alignment was checked and since this original alignment had gaps (Additional file 4: File S2), all nucleotides in the respective column which had a gap were manually removed by deleting this column until no gap remained in the complete final alignment. In addition, positions (i.e., columns within the alignment) in which ambiguities were present, were manually deleted as well. There were a total of 7721 positions in the final dataset (Additional file 5: File S3). MEGA6.06 allows precomputation of the best nucleotide substitution model for Maximum Likelihood (ML) to match the data, including General Time Reversible, Hasegawa-Kishino-Yano, Tamura-Nei, Tamura 3-parameter, Kimura 2-parameter, and Jukes-Cantor. Non-uniformity of evolutionary rates among sites may or may not be modeled by using a discrete Gamma distribution (+G) with 5 rate categories and by assuming that a certain fraction of sites are evolutionarily invariable (+I). The model with the lowest Bayesian Information Criterion score is considered to best describe the substitution pattern [62], which is General Time Reversible with gamma distribution and invariable sites in this case. For the precomputation, the tree topology was automatically computed using Neighbor-Joining.

The final evolutionary history was inferred by using the ML method based on the General Time Reversible model. The tree with the highest log likelihood (−58141.5) is shown. Values within the tree indicate the percentage of 1000 bootstrap tests showing the same cluster. Bootstrap values below 50 % are not shown. Initial trees for the heuristic search were obtained by applying the Neighbor-Joining method to a matrix of pairwise distances estimated using the Maximum Composite Likelihood (MCL) approach. A discrete Gamma distribution was used to model evolutionary rate differences among sites (5 categories, parameter = 0.6072). The rate variation model allowed for some sites to be evolutionarily invariable. The final tree is drawn to scale, with branch lengths measured in the number of substitutions per site.

## Results

### Discovery of an overlapping protein-coding ORF displaying protein-coding features

**Fig. 1:**
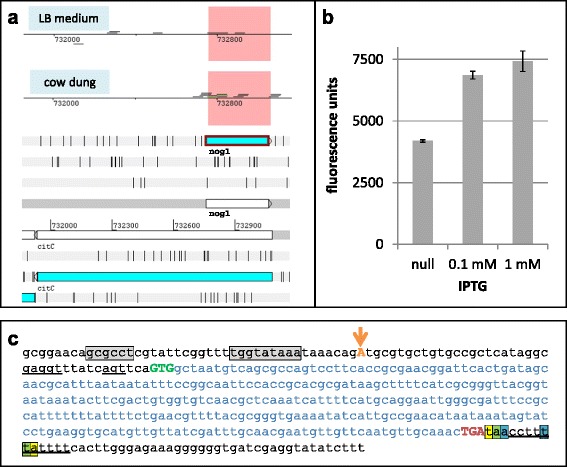
**a** Strand-specific transcription signals of *nog1*. Shown are the transcriptome data of EHEC (upper panel) grown aerobically in 1:10 diluted LB medium (upper line) or incubated in cow dung (lower line). The transcriptome sequencing reads are shown above or below the genome line for the forward and reverse strand respectively. Only a single read of *citC* is visible in the LB-condition. However, *nog1* (pink shaded area) is induced about 14-fold in cow dung compared to LB, based on RPKM values. The lower panel shows the genomic architecture around *citC*, drawn using Artemis [93]. The open reading frames of *nog1* and *citC* are indicated by blue arrows in the respective reading frames. **b** Fluorescence units of *nog1* C-terminally fused with *gfp* after IPTG induction for 2 h using the indicated inducer concentrations. This experiment shows that *nog1* is principally able to be translated by ribosomes. **c** Genetic organization of *nog1*. Grey, predicted promoter (BProm); orange letter, transcription start site according to the transcriptome sequencing data (Fig. 1a); orange arrow, transcription start site according to 5’-RACE; underlined, possible Shine-Dalgarno sequences; green, predicted start codon; blue, coding sequence; red, stop codon; bases highlighted by bold underline, predicted rho independent terminator with possible hairpin structure (WebGeSTer)

In previous experiments, EHEC was grown under eleven different growth conditions and strand specific transcriptomes were sequenced [27]. These conditions comprised LB medium at pH4, pH7, pH9, or at 15 °C; LB with addition of nitrite or trimethoprim-sulfamethoxazole; LB-agar surface, M9 minimal medium, spinach leaf juice, the surface of living radish sprouts, and cow dung. In the condition “cow dung”, we found a novel RNA to be induced about 14-fold compared to LB, based on RPKM values (Fig. 1a). Briefly, for the condition “cow dung”, 10 g cattle feces were inoculated for 6 h at 37 °C with EHEC pre-grown in LB [27]. This RNA covers an overlapping ORF which consists of 306 bp (position from 732757 to 733062 in EHEC genome, accession no. NC_002655) and is completely embedded in antisense to *citC* in frame -2 (position 733079 to 731934, the reading frame of *citC* being defined as +1; Fig. 1a). Thus, we wondered whether this novel RNA was maybe protein coding and undertook experiments to verify this hypothesis. To facilitate further reading, we introduce the suggested gene name *nog1* here (*n*ovel *o*verlapping *g*ene).

The novel ORF *nog1* was C-terminally fused in frame to *gfp* to determine protein production. After induction, GFP fluorescence was increased compared to the uninduced control showing that the novel ORF *nog1* is indeed translatable (Fig. 1b). However, we could not detect Nog1 induced from its natural promoter and fused to His and Myc tags (data not shown). Despite enrichment using Ni-columns and acetone precipitation, His-Myc-tagged Nog1 was not detectable in Western blots using Myc-tag antibodies. But acetone precipitation is prone to lose small proteins and Nog1 is predicted to be unstable and a membrane protein (see below), which may have rendered immunological detection of this short protein difficult. However, the above *nog1*::*gfp* fusion shows that the ribosomes pass the GTG start codon to translate the *nog1* ORF.

Next, we predicted promoter, Shine-Dalgarno and terminator sequences for *nog1*, all of which were found (Fig. 1c). A possible transcription start site in the vicinity of the predicted promoter could be the read mapping farthest upstream in the strand-specific transcriptome sequencing (Fig. 1a). The read starts 40 bp upstream of the putative start codon. To verify this position, the +1 was determined using 5’-RACE. The latter result is in perfect agreement with the former since the indicated transcription start site of the new ORF was also found to be 40 bp upstream of the putative start codon GTG. Thus, this position is suggested to be the major +1 for *nog1* (Fig. 1c, orange arrow).

### Promoter activities of the genomic regions upstream of *nog1* and *citC*

**Fig. 2:**
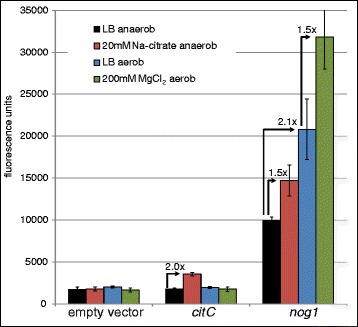
Fluorescence measurements of promoter::*gfp* fusions for empty vector control (*left*), P_*citC*_ (*middle*) and P_*nog1*_ (*right*). The fluorescence has been normalized to OD_600nm_ and the conditions are indicated

The promoter region immediately upstream of *nog1* was fused to *gfp* and tested for inducibility. Unfortunately, cow dung was not usable for these experiments due to the opaqueness, autofluorescence, and the many other bacteria present in this substrate. Sterile filtration before and after dilution was attempted, but extremely difficult due to the high content in fine particulate matter. To ease analysis, we decided to test LB medium supplemented with a number of various inorganic and organic stressors in sub-inhibitory concentrations for promoter activity (see Methods). Using LB medium as a control, promoter P_*nog1*_ activity was increased when cells were grown aerobically in the presence of 320 μM menadione (3-fold), 5 mM malonic acid (2.5-fold), and 150 mM magnesium chloride (1.5-fold; Fig. 2). The transcription of *citC* is only induced in the presence of citrate under anaerobic conditions [63], thus, these conditions were tested additionally (Fig. 2). P_*citC*_ becomes active about 2-fold when bacteria are grown anaerobically with the addition of 20 mM citrate. Interestingly, P_*nog1*_ activity increased under anaerobiosis in plain LB, as well as in citrate supplemented LB (Fig. 2), and, in addition, when adding menadione (2-fold) or malonic acid (5-fold).

### Metabolome of the translationally arrested mutants compared to the wild type

**Fig. 3:**
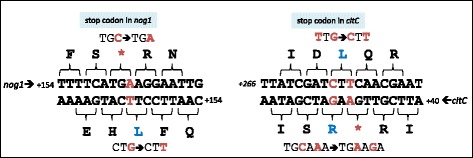
Mutations introduced to arrest the translation of *nog1* (*left*) or *citC* (*right*) strand specifically. The numbers indicate the distance of the sequence shown from the start codons of *nog1* and *citC* respectively. Note that the amino acid sequence of the reading frame in antisense remains unchanged in both cases. The positions of the mutations are shown in red

To uncover phenotypes related to the gene product Nog1, its translation was arrested by introducing a stop codon without changing the amino acid sequence of the mother reading frame *citC* (Δ*nog1*, Fig. 3, left). Similarly, *citC* was arrested without changing Nog1 (Fig. 3, right). If *nog1* were being a non-coding RNA, its function would not be disturbed by a single nucleotide exchange. In contrast, translation is arrested by the artificial stop codon and Nog1 production ceases. The metabolome of the mutant compared to wild type was analyzed by non-targeted profiling approaches using either gas chromatography coupled with mass spectrometry (GC-MS) or ion cyclotron resonance Fourier transform mass spectrometry (ICR-FT/MS).

For GC-MS, six biological replicates of both wild type and Δ*nog1* were grown in plain LB and significantly different metabolites determined (*p* ≤ 0.05). Only a decrease of metabolites in the *nog1*-mutant was found. For instance, tryptophan had the highest fold decrease of about 4.7. Next were citric and isocitric acid, as well as succinate with a decrease of about 1.5 to 2-fold (further metabolites are in Additional file 6: Table S3). Hence, we believe there is a slight influence of the Δ*nog1* mutation on the primary metabolism, probably with a focus on the TCA cycle due to changes in citrate and succinate.

Using the more sensitive and accurate ICR-FT/MS for profiling, several thousand features were detected (data not shown) and MassTRIX [34] annotated several hundred metabolites from different pathways. Significantly different molecules between wild type and mutant (*p* ≤ 0.05) included metabolites, e.g., of the glutathione metabolism (glutathione decreased 5.6-fold in Δ*nog1* compared to wild type). Furthermore, amino acid metabolism pathways were affected, since corresponding metabolites were found to be changed (see Additional file 7: Table S4). The latter fits with the observed change in tryptophan levels in the GC-MS experiment above. GC-MS and ICR-FT/MS complement each other. ICR-FT/MS is not able to detect small masses, e.g., of tricarboxylic acid cycle (TCA) intermediates, but glutathione metabolites were not identifiable in the GC-MS experiment. However, targeted investigations are needed to further support the above results since only a weak metabolic phenotype is found in both assays.

### Fitness of translationally arrested Nog1 and CitC mutants

**Fig. 4:**
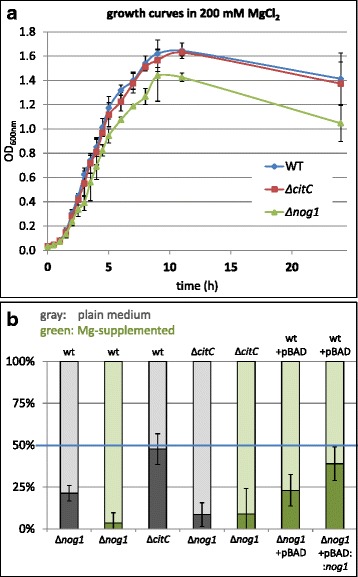
Growth phenotype of wild type, and Δ*citC* and Δ*nog1.* **a** Growth curves of wild type, and Δ*citC* and Δ*nog1* strains grown separately in Schott bottles under shaking at 37 °C. The average OD values and the standard deviations from three replicates are given. **b** Percentage of strains after growth in competitive growth assays. Gray columns indicate plain LB medium, green columns LB supplemented with MgCl_2_. The experiments were repeated at least three times and standard deviations are shown. First column, growth of Δ*nog1* against wild type in LB; second column, Δ*nog1* against wild type in LB with MgCl_2_; third column, growth of Δ*citC* against wild type in LB; fourth column, growth of Δ*nog1* against Δ*citC* in LB; fifth column, growth of Δ*nog1* against Δ*citC* in LB with MgCl_2_; sixth column, growth of Δ*nog1* + pBAD competing against wild type + pBAD in LB with MgCl_2_; seventh column, Δ*nog1* + pBAD::*nog1* was grown together with wild type + pBAD in LB with MgCl_2_. The last two experiments were performed in the presence of ampicillin and arabinose

When wild type and Δ*nog1* mutant were grown separately under a variety of stress conditions, no differences in growth rates were observed (data not shown), except a slightly reduced growth of the Δ*nog1* mutant in medium supplemented with MgCl_2_ (Fig. 4a). In contrast, competitive growth assays comparing two strains (e.g., mutant versus wild type) in the same flask are highly sensitive tools to detect even small fitness differences between two strains [64, 65]. For competition experiments, the two strains were mixed in equivalent small cell numbers (50:50) and broth supplemented with MgCl_2_ at sub-inhibitory concentration was inoculated using the strain mixture. A change in relative fitness between the two strains becomes apparent by determining the fraction of one strain over the other. After 18 h of aerobic growth, the performance of each strain was measured (Fig. 4b). Interestingly, Δ*nog1* shows a clear loss of fitness compared to its parental strain when grown in plain LB. When using MgCl_2_-supplemented broth, the decrease in fitness of *Δnog1* was even more pronounced, corroborating the finding of retarded growth in MgCl_2_-supplemented medium (compare to Fig. 4a). A mutant translationally arrested in *citC*, however, did not show any fitness differences when competitively grown against its parental strain (Fig. 4b). Accordingly, when Δ*nog1* was grown competitively against Δ*citC* a similar decrease in fitness was observed comparable to the wild type strain, both in plain LB and medium supplemented with MgCl_2_.

For complementation experiments, the *nog1* ORF was cloned downstream of an arabinose inducible promoter in the plasmid pBAD. At first, the translationally arrested Δ*nog1* mutant and wild type, both transformed with an empty pBAD, were grown competitively in MgCl_2_-supplemented medium. Unexpectedly, the difference in competition, visible when using vector-less strains in this medium, diminished somewhat. Next, Δ*nog1* was transformed with pBAD::*nog1* to compete against the wild type containing an empty pBAD. The Δ*nog1* strain containing pBAD::*nog1* competed better against wild type containing the pBAD vector, although it could not be fully restored (Fig. 4b). We hypothesize that the indispensable addition of ampicillin and arabinose to the latter experiments disturbed the competition experiments to some extent.

### Bioinformatics analysis of the Nog1 protein sequence

The new overlapping-encoded protein Nog1 is predicted to have 101 amino acids, a theoretical molecular weight of 11.15 kDa, a pI of 11.36, an aliphatic index of 117.72, and the GRAVY value is 0.564 [66]. No signal peptide or domain was predicted [38, 59]. The instability index was computed to be 42.05 which classifies the protein as being unstable [58]. Secondary structures and membrane domains were predicted, using a number of similar programs allowing comparison of the results for different algorithms. This assumes that secondary structures predicted by several programs are possibly more reliable. The Nog1 protein structure potentially consists of three short helices and several very short β-sheets. At least one membrane domain was predicted by all algorithms used (compare to Additional file 8: Figure S1). The protein may be localized in the inner membrane according to the software LocTree3 [60], most likely with the N-terminus outside and the C-terminus inside, as agreed on by two of three programs for transmembrane topology prediction [42, 43].

### Phylogenetic analysis of *nog1*

**Fig. 5:**
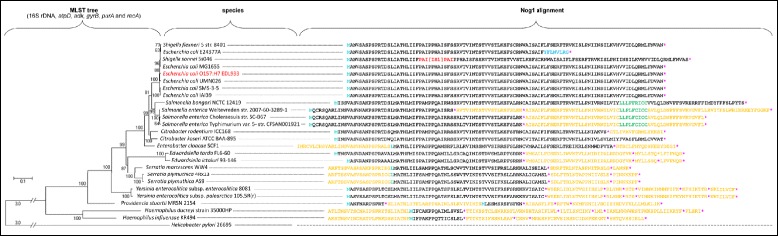
Phylogenetic tree of *nog1*-bearing *E. coli* and *Shigella* strains and species carrying *nog1*-like sequences. On the left, phylogenetic tree of representative bacterial strains containing a *nog1* or *nog1*-like open reading frame within *citC*. The tree was inferred using the Maximum Likelihood method and is based on a concatemer of 16 s RNA, *atpD*, *adk*, *gyrB*, *purA*, and *recA*, thus, independent of *citC* and *nog1* respectively. The percentage of trees which clustered together in 1000 bootstrap replicates is shown next to the branches. *Helicobacter* was used as out-group. On the right, an alignment of *nog1* and *nog1*-like sequences within *citC* of the strains is shown. Black, sequence parts which can be aligned to *nog1* of EHEC using BLAST2 and which might be translated; turquoise, N-terminal methionine; pink, translational stop; blue, frame shift mutation (which destroyed both, *nog1* and *citC*); red, insertion of IS1 and duplicated amino acids ProAlaIle around the insertion site; this insertion also destroys *nog1* and *citC*. Orange, regions likely not to be translated; green, insertion element in some *Salmonella* and *Citrobacter* strains, keeping the frames intact

In order to elucidate the phylogeny of *nog1*, a phylogenetic tree including EHEC was constructed of species harboring *citC* (only a representative subset was used omitting identical or very similar sequences) with *Helicobacter* as an out-group (Fig. 5). The phylogenetic tree calculation was independent of *citC* since it was based on a concatemer of 16S *rDNA*, *atpD*, *adk*, *gyrB*, *purA* and *recA*. All six genes belong to the core genome of the species included in the analysis. Homologs of *citC* were then analyzed for the presence of *nog1* in their −2 antisense reading frame. The full reading frame *nog1* was found in *Escherichia* or *Shigella*. In a few instances, its reading frame was destroyed, for example due to an insertion sequence or a frame shift which also destroyed *citC* (the only two of such species found are shown in Fig. 5). Several *Salmonella* species contain a *nog1*-like part in their *citC* sequence. Depending on the *Salmonella* strain, *nog1* appears to be either elongated or fragmented, but whether *nog1* is indeed functional in some *Salmonella* strains remains unknown. In all other genera, the *nog1* reading frame is clearly fragmented (Fig. 5).

## Discussion

### *nog1* probably encodes the protein Nog1

In this paper we report that *nog1* possesses an active promoter, activated in natural as well as lab conditions (Figs. 1a, and 2). A transcription start was determined, the gene is followed by a terminator sequence (Fig. 1c) and a protein can be expressed (Fig. 1b). In addition, we provided ample evidence that the ∆*nog1* mutant shows a weak phenotype, especially in MgCl_2_-supplemented medium (Fig. 4; Additional file 6: Table S3; Additional file 7: Table S4). This data clearly shows that the gene is functional, although of apparently minor influence to the fitness of *E. coli*.

We were successful in demonstrating a stable gene product at the protein level when fusing Nog1 to GFP and detecting the latter by its fluorescence (Fig. 1b) but one could still hypothesize, despite this finding, that *nog1* actually may encode a novel ncRNA rather than a protein *in vivo*. A number of relatively short RNAs are not translated and might function solely as ncRNAs at the RNA level [67, 68]. However, we suggest rejecting the “non-coding RNA hypothesis” of *nog1* for the following five reasons.

First, a bioinformatics analysis of the open reading frame of *nog1* (Fig. 1c, Additional file 8: Figure S1) shows clear characteristics of a protein encoding ORF, including a ribosome binding site in proper distance from the start codon. While it cannot be excluded that such sequence characteristics may occur just by chance, we consider this to be a remote option [69]. Second, if *nog1* regulates *citC* via antisense silencing, *nog1* should only be induced when *citC* mRNA is present [70]. However, *nog1* is induced in cow dung where no transcript of *citC* could be detected (Fig. 1a). Promoter studies of this and other work show that *citC* is expressed only under anaerobic conditions, but *nog1* was also detectable in aerobic conditions. Thus, it is quite unlikely that *nog1* acts as regulatory RNA in an anti-sense fashion against *citC* since, for antisense-RNA regulation, the presence and base pairing of both sense and antisense transcripts is required [71]. Third, the introduction of a single base pair change only, to translationally arrest *nog1*, leads to a clear phenotype which would not be expected when *nog1* is an antisense regulator. Also, a hypothetical ribozyme activity would require stable base pairing involving many nucleotides [72]. Single base pair changes, such as those used in this study to translationally arrest *nog1* (Fig. 3), are unlikely to cause detectable differences in an RNA-RNA pairing event which involves many base pairs. Fourth, a translationally arrested Δ*citC* mutant which introduced a nucleotide change synonymous in the overlapping *nog1* did not, as was expected, display any fitness differences in competitive growth experiments (Fig. 4). Finally, supplementing the Δ*nog1* strain with a functional *nog1* on a plasmid *in trans* means that Nog1 is produced, and the strain competes somewhat better against wild type in MgCl_2_-supplemented medium (Fig. 4b). However, if *nog1* acts as ncRNA, this strain should compete even less against the wild type since the gene dosage of the plasmid-borne gene is higher and more ncRNA would be present to regulate its targets.

### Potential function of Nog1

Transcription of *nog1* is specifically induced in cow dung (Fig. 1a), and the promoter is activated by magnesium ions (Fig. 2), menadione and malonic acid. In addition, magnesium ions had an effect on growth of Δ*nog1* in growth and competitive growth assays (Fig. 4). However, the function of *nog1* remains cryptic. It would have been interesting to elucidate the function of *nog1* in cow dung further, but this is a difficult substrate to work with. For this reason we relied on lab media. Changes in metabolite concentrations of the Δ*nog1* mutant are small compared to wild type in plain LB, so *nog1* is only slightly involved in any central metabolic reaction (e.g. TCA cycle, amino acid metabolism; Additional file 6: Table S3, Additional file 7: Table S4). Since the ∆*nog1* mutant displayed a small growth disadvantage in MgCl_2_-supplemented medium (Fig. 4a), bacteria carrying *nog1* may have an increased fitness in environments reflecting this or other unknown conditions. Whatever function this gene exerts, its modificatory action is at least strong enough to cause a phenotype in a competitive growth assay. Since, according to the bioinformatics analysis, Nog1 is membrane bound, it could either act as sensor or modificatory protein in the membrane. However, further experiments would have to support this hypothesis.

### Recent origin of *nog1* by overprinting

The citrate-lyase ligase gene *citC*, which is the mother gene of *nog1*, is taxonomically broadly distributed (mostly in γ-proteobacteria and firmicutes, but also in some other bacteria). In contrast, *nog1* is found only in the *Escherichia*/*Salmonella* clade. Such a taxonomical distribution could be explained by an ancient overprinting event followed by many subsequent deletions of the *nog1* reading frame in all clades except the *Escherichia*/*Salmonella* clade. However, for parsimony reasons we suggest a recent origin of *nog1* after the separation of *Escherichia*/*Salmonella* from the rest of the enterobacteria (Fig. 5).

Based largely on research in eukaryotes, recently arisen genes are thought to be shorter and less important compared to genes with a longer evolutionary history [11, 73, 74]. After a short evolutionary history, novel genes should be less well-integrated in the cellular metabolism and, therefore, may provide only a limited fitness gain for the cell. Since young genes are expected to be not yet well-adapted, due to their short evolutionary history, they may be expressed at a low level only. New genes should be orphans [75] and overlapping genes have been proposed to be phylogenetically more restricted than the mother gene they overlap [17]. Finally, *de novo* formed genes may have a strain specific function only [76], which could be important for niche specific adaptations assumed for orphan genes [77].

We suggest that *nog1*, embedded completely in its mother frame *citC*, offers a number of characteristics which fit the hypothesis of *nog1* being a young gene: (i) *nog1* shows a restricted occurrence within the closely related genera of *Escherichia*/*Salmonella*, (ii) the gene is short, (iii) it is weakly expressed, (iv) it appears to be only marginally associated in *E. coli*’s central metabolism, and (v) the gene product provides only a small fitness advantage for its carrier. Conversely, the mother frame *citC* is widely distributed among many bacterial species since it is important to metabolize citrate (in the TCA) under anaerobic growth conditions [28]. Thus, we hypothesize that *nog1* may have arisen by a recent overprinting event, probably after the split of the *Escherichia*/*Shigella* clade or after the divergence of *Escherichia*/*Salmonella* from the other γ-proteobacteria (Fig. 5).

## Conclusions

According to Nekrutenko & He [78] “[…] genes with overlapping reading frames exemplify some of the most striking biological phenomena […]”. This is a statement with which we agree, especially since Johnson and Chisholm [14] proposed an information content constraint which should prevent non-trivially overlapping genes to form freely. Indeed, the initial sequence features of the *citC* mother reading frame, which would allow for a successful overprinting process producing a Nog1 protein with at least a weak initial function, are unknown and certainly require further investigation.

Bacteriological research has never been directed to discover overlapping genes. On the contrary, bacterial genome annotation programs systematically remove overlapping genes [73, 79–81], thereby efficiently preventing their discovery. Perhaps it is not surprising that with the exception of *E. coli*’s three overlapping genes, only a few further examples have been discovered rather accidentally in *Streptomyces* [82], *Pseudomonas* [83, 84] or *Xanthomonas* [85]. Functional analyses of overlapping genes in bacteria are even rarer.

### Bacterial overlapping genes may be more common than expected

Four lines of argument lead us to suggest that overlapping genes in bacteria are more common than is currently assumed by microbiologists.

First, a systematic bioinformatics analysis of bacterial overlapping ORFs demonstrates that bacterial genomes contain a larger number of long overlapping ORFs than is expected based on a statistical analysis [86]. Random mutational drift would have eliminated this signal long ago if no selection pressures were stabilizing these ORFs. Indeed, Sabath, et al. [76] found that overlapping loci are under weak positive selection.

Second, during various comprehensive transcriptomic analyses of *E. coli* targeted to its differential gene expression in different habitats [27], we have noticed that many overlapping reading frames show a transcriptional signal which is clearly above background and is probably not due to ncRNA (data not shown). Such signals might be evidence for the existence of further overlapping genes.

Third, a bioinformatics analysis suggests that overlapping genes appear abundantly in some viruses [87, 88]. There is good evidence that all bacteria are parasitized by viruses and bacteriophage vastly outnumber bacterial cells on earth [89]. Furthermore, genomes of bacterial viruses are constantly being mixed, by various mechanisms, with the genomes of their hosts, which should result in an increase of overlapping genes in bacteria [90].

Fourth, for eukaryotes it has been suggested that completely new genes evolve frequently *de novo* from non-coding DNA and for a few cases even overprinting has been suggested [5, 10, 73]. Since prokaryotic genomes are densely packed with established genes [91], that source for novel genes is almost absent and we suggest overprinting as a more important alternative to gene duplication for these organisms acquisition of completely novel genes. This exploits a huge hypothetical hidden coding reserve potentially providing a “novelty pool“ for adaptation [92].

While in this paper we have presented specific experimental evidence for the existence of a fourth overlapping gene in *E. coli* EHEC, we suggest that this may be an initial finding only. For the origin of gene novelties in bacteria, such cases of new overlapping genes could lead to the establishment of overprinting as a potentially more significant alternative to gene duplication, once microbiologists and evolutionary biologists start to target experimental research along this research path.

### Availability of supporting data

All data and protocols supporting the results of this article are available in the repository labarchives.com (https://mynotebook.labarchives.com/) using the links https://doi.org/10.6070/H42N5083, https://doi.org/10.6070/H4XW4GSD, https://doi.org/10.6070/H4T43R3V, https://doi.org/10.6070/H4PG1PR0, https://doi.org/10.6070/H4JQ0Z24, https://doi.org/10.6070/H4DZ06BS, https://doi.org/10.6070/H4959FKC, and https://doi.org/10.6070/H45D8PVN. All the supporting data are included as additional files.

## Additional files

Additional file 1: Table S1.
*E. coli* strains and plasmids. (PDF 116 kb) Additional file 2: File S1. Full description of the metabolomics measurements. (PDF 125 kb) Additional file 3: Table S2. Accession numbers and genes used for the phylogenetic tree. (XLSX 154 kb) Additional file 4: File S2. Alignment of the concatenated sequences used for tree construction before gap deletion. (TXT 221 kb) Additional file 5: File S3. Alignment of the concatenated sequences with gaps deleted. (TXT 196 kb) Additional file 6: Table S3. Full data set of the gas chromatography coupled with mass spectrometry (GC-MS). (XLSX 20 kb) Additional file 7: Table S4. Full data set of the ion cyclotron resonance Fourier transform mass spectrometry (ICR-FT/MS). (XLSX 15 kb) Additional file 8: Figure S1. Overview of bioinformatics predictions for Nog1. (PDF 110 kb)

## Abbreviations

5’-RACE: 5’ rapid amplification of cDNA ends
AP: Alkaline phosphatase
BCIP: 5-bromo-4-chloro-3-indolylphosphate
blast: Basic local alignment search tool
bp: Base pairs
BSA: Bovine serum albumin
EHEC: Enterohemorrhagic *E. coli*
GC-MS: Gas chromatography coupled with mass spectrometry
GFP: Green fluorescent protein
GRAVY: Grand average of hydropathy
ICR-FT/MS: Ion cyclotron resonance Fourier transform mass spectrometry
IPTG: Isopropyl-β-D-thiogalactopyranosid
LB: Luria-Bertani medium
MCL: Maximum composite likelihood
ML: Maximum likelihood
MS Excel: Microsoft Excel
Nal: Nalidic acid
NBT: Nitroblue tetrazolium
ncRNA: Non-coding RNA
NEB: New England Biolabs
*nog1*: Novel overlapping gene
OD_600 nm_: Optical density at 600 nm wavelength
ORF: Open reading frame
PBS: Phosphate-buffered saline
PCR: Polymerase chain reaction
PVDF: Polyvinylidene difluoride
Q_3_: Three-state description of α-helix, β-sheet and coil
RPKM: Reads per kilobase per million mapped reads
rpm: Revolutions per minute
SDS: Sodium dodecyl sulfate
TCA cycle: Tricarboxylic acid cycle
Tris: 2-amino-2-hydroxymethyl-propane-1,3-diol
T‑test: Student’s test
