# Supplementary material for: Evidence for the recent origin of a bacterial protein-coding, overlapping orphan gene by evolutionary overprinting
Source: BMC Evol Biol. 2015 Dec 18;15:283. doi: 10.1186/s12862-015-0558-z (PMC4683798; doi:10.1186/s12862-015-0558-z)
Supplement: Additional file 1: Table S1. — E. coli strains and plasmids. (PDF 116 kb) [file 12862_2015_558_MOESM1_ESM.pdf]

Table S3. *E. coli* strains and plasmids

**Strain or plasmid**	**Relevant characteristics**	**Reference**
E. coli O157:H7 EDL933 (EHEC)	wild type, outbreak strain, obtained from Collection de l´Institute Pasteur (CIP 106327)	(Perna *et al.*, 2001)
EHEC Nal^R	spontaneous nalidic acid resistant clone of EHEC	this work
E. coli SM10λpir	*Th*-1*thr leu* tonA *lacY supE*, *recA*::RP4-2-Tc::Mu Km^R (λ pir)	(Miller & Mekalanos, 1988)
E. coli CC118 λpir	*araD139* Δ(*ara,leu*)7697 Δ*lacX*74 Δ*phoA20 galK thi rpsE rpoB argE(Am) recA*1 (λ pir)	(Manoil & Beckwith, 1985)
pMRS101	*oriR6K strAB mobRK2 sacB bla ori*E1	(Sarker & Cornelis, 1997)
pProbe-NT	pBBR1 replicon, *gfp* reporter, Km^R	(Miller *et al.*, 2000)
pBAD/*Myc-His*	pBR322-derivative, *araBAD* promoter, *myc C*-tag and his-tag fusion	Invitrogen

References

Manoil, C. & J. Beckwith, (1985) TnphoA: a transposon probe for protein export signals. *Proc Natl Acad Sci U S A* **82**: 8129-8133.

Miller, V. L. & J. J. Mekalanos, (1988) A novel suicide vector and its use in construction of insertion mutations: osmoregulation of outer membrane proteins and virulence determinants in *Vibrio cholerae* requires *toxR*. *J Bacteriol* **170**: 2575-2583.

Miller, W. G., J. H. Leveau & S. E. Lindow, (2000) Improved *gfp* and *inaZ* broad-host-range promoter-probe vectors. *Mol Plant Microbe Interact.* **13**: 1243-1250.

Perna, N. T., G. Plunkett, 3rd, V. Burland, B. Mau, J. D. Glasner, D. J. Rose, G. F. Mayhew, P. S. Evans, J. Gregor, H. A. Kirkpatrick, G. Posfai, J. Hackett, S. Klink, A. Boutin, Y. Shao, L. Miller, E. J. Grotbeck, N. W. Davis, A. Lim, E. T. Dimalanta, K. D. Potamousis, J. Apodaca, T. S. Anantharaman, J. Lin, G. Yen, D. C. Schwartz, R. A. Welch & F. R. Blattner, (2001) Genome sequence of enterohaemorrhagic *Escherichia coli* O157:H7. *Nature* **409**: 529-533.

Sarker, M. R. & G. R. Cornelis, (1997) An improved version of suicide vector pKNG101 for gene replacement in gram-negative bacteria. *Mol Microbiol* **23**: 410-411.
