# Supplementary material for: Evidence for the recent origin of a bacterial protein-coding, overlapping orphan gene by evolutionary overprinting
Source: BMC Evol Biol. 2015 Dec 18;15:283. doi: 10.1186/s12862-015-0558-z (PMC4683798; doi:10.1186/s12862-015-0558-z)
Supplement: Additional file 2: File S1. — Full description of the metabolomics measurements. (PDF 125 kb) [file 12862_2015_558_MOESM2_ESM.pdf]

FULL DESCRIPTION OF THE METABOLOMICS MEASUREMENTS

Metabolomes by GC-MS

The methanol soluble part of the metabolome was determined as follows. Six biologically independent cultures of mutant and six wild-type cultures were inoculated and grown on the same day using a highly standardized protocol, e.g., the same batch of growth medium. Briefly, biological replicate cultures were grown shaking (150 rpm) in 20 mL of 1:2 diluted LB-medium in bottles at 37°C to OD_{600nm} = 1. Four mL of bacterial culture were rapidly vacuum filtered using a HVLP filter with a pore size of 0.45 µm and a diameter of 4.6 cm (Millipore). Filters were flash frozen in liquid nitrogen. About 100 µL of 0.1 mm and few 2 mm zirconia beads, 1 mL ice cold methanol was added. Bacteria were extracted in a FastPrep™-24 (MP Biomedicals) at 6.5 m/s for 45 s for three times. Between runs, the samples were cooled in liquid nitrogen (stopped before freezing) to avoid excessive heating during liberation. The samples were centrifuged (14,000xg, 4°C, 3 min) and supernatants were transferred into new tubes and dried using a centrifugal vacuum concentrator (SPD 111V SpeedVac, Thermo Savant, New York). Dried samples were derivatized for metabolite analysis via GC-MS in a two-step procedure as described [1]. First, carbonyl moieties were protected via methoximation with 20 µL of 40 mg/mL methoxyamine hydrochloride in pyridine (90 min, 30°C). Afterwards, acidic protons were derivatized by adding 30 µL MSTFA (N-methyl-N-trimethylsilyltrifluoroacetamide) and an incubation step for 40 min at 60°C. For determination of retention time index, 1 µL of the retention time index marker cocktail (n-alkanes ranging from C_{10} to C_{36}, each 200 µg/mL in pyridine) were added to each sample. For metabolite profiling a HP Agilent 7890 gas chromatograph was used to perform GC analysis and coupled to an Agilent 5975 Quadrupole mass spectrometer (Agilent Technologies, Böblingen) for mass determination. Samples were injected by a volume of 1 µL in splitless mode and separation was performed on a VF-5ms capillary column, 30 m in length (0.25 mm inner diameter, 25 µm film thickness; VARIAN, Palo Alto) at a constant flow of 1 mL per minute in helium. Temperature started isocratic with 1 min at 70°C and was progressively ramped at 10°C per minute up to 330°C which was held for 8 min. Mass analysis was performed by scan rates of 2.5 per s,

and mass ranges detected between 40 to 600 Da. The software MetaboliteDetector (version 2.06) was used for processing all chromatograms. This included automated baseline correction, peak finding, area calculation, library search and deconvolution of all chromatogram mass spectra and mass-spectral correction for co-eluting metabolites. Retention indices (RI) were calculated and suitable fragment mass-to-charge ratios for selective quantification were identified. In order to identify metabolites RI and defined spectra libraries (Golm Metabolome Database and in house library) were used. For peak area quantification, three selected fragment ions specific for each individual metabolite were used. Finally, each compound was normalized by the peak area of the internal standard and obtained relative response ratios could be compared without knowledge of absolute compound concentrations. For an experimental setting all determined peaks for each chromatogram were aligned. After alignment, deconvoluted peaks of each chromatogram were filtered via 70% reproducibility and exclusion of systemic peaks. Differences in the metabolome between wild type and the mutants were tested for significance using the Student test (T-test; $p \leq 0.05$).

DETERMINATION OF METABOLOMES ICR-FT/MS

Metabolite profiling was carried out using Ion cyclotron resonance Fourier transform Mass spectrometry (ICR-FT/MS) on a Bruker solariX equipped with a 12 T magnet (Bruker Daltonics, Bremen). Three biological replicate cultures of each wild type and Δ*nog1* mutants were grown, filtered and frozen as above. Bacteria were extracted with 50% methanol using the FastPrep as above. Samples were centrifuged and supernatant was filtered using a 0.22 µm pore size filter to remove any larger particles. Samples were diluted 1:20 with 70% methanol prior to analysis. ICR-FT/MS was externally calibrated on clusters of arginine (10 ppm in 70% methanol). A time domain transient of 2 megawords was used and 300 scans were accumulated for one spectrum. Samples were infused with syringe pump at a flow rate of 120 µL/h. For data analysis, spectra were internally calibrated with an error smaller 0.1 ppm. Spectra were exported with a signal-to-noise ratio of 3 and aligned within a 1 ppm window using in-house developed software. Putative metabolites were

annotated using the MassTRIX webserver [2]. Masses in less than 2 replicates of a group were removed. Statistical analysis was carried out in MS Excel 2010 and Genedata Expressionist for MS 7.6 (Genedata, Martinsried) using Welch's T test ($p \leq 0.05$) [3, 4].

References

1. Scherling C, Roscher C, Giavalisco P, Schulze ED, Weckwerth W: **Metabolomics unravel contrasting effects of biodiversity on the performance of individual plant species**. *PloS one* 2010, **5**(9):e12569.
2. Wägele B, Witting M, Schmitt-Kopplin P, Suhre K: **MassTRIX reloaded: combined analysis and visualization of transcriptome and metabolome data**. *PloS one* 2012, **7**(7):e39860.
3. Xia J, Mandal R, Sinelnikov IV, Broadhurst D, Wishart DS: **MetaboAnalyst 2.0--a comprehensive server for metabolomic data analysis**. *Nucleic Acids Res* 2012, **40**(Web Server issue):W127-133.
4. Saeed A, Sharov V, White J, Li J, Liang W, Bhagabati N, Braisted J, Klapa M, Currier T, Thiagarajan M: **TM4: a free, open-source system for microarray data management and analysis**. *BioTechniques* 2003, **34**(2):374.
