# Supplementary material for: Evidence for the recent origin of a bacterial protein-coding, overlapping orphan gene by evolutionary overprinting
Source: BMC Evol Biol. 2015 Dec 18;15:283. doi: 10.1186/s12862-015-0558-z (PMC4683798; doi:10.1186/s12862-015-0558-z)
Supplement: Additional file 8: Figure S1. — Overview of bioinformatics predictions for Nog1. (PDF 110 kb) [file 12862_2015_558_MOESM8_ESM.pdf]

Suppl. Fig. S1

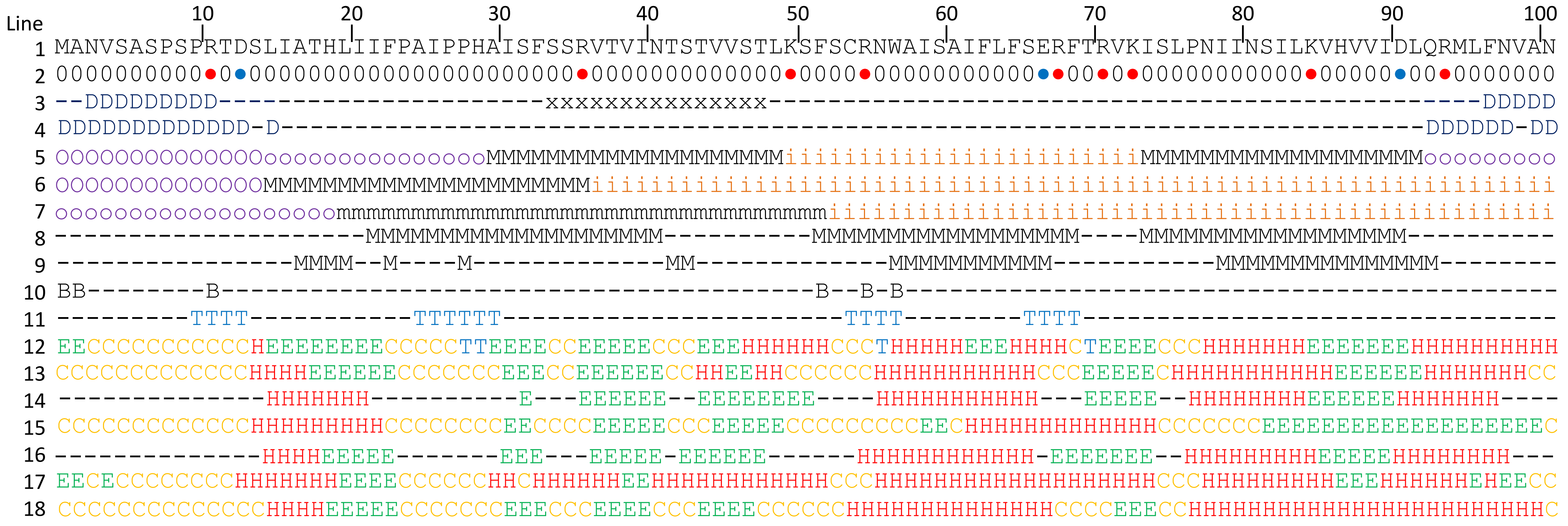

Line 19
B Protein binding site

Line 1
Protein sequence

Line 2
0 Uncharged
● Positive charge
● Negative charge

Lines 3 - 4
D Disordered region
x Low complexity region

Lines 5 - 9
O/o Outside (high / low probability)
I/i Inside (high / low probability)
M/m Membrane (high / low probability)
B Protein binding

Line 10
B Protein binding site

Lines 11 – 18
H Helix
E Sheet
C Coil
T Turn

Fig. S1. Overview of bioinformatic predictions for Nog1.
Lines: The numbers correspond to the line shown in the figure:

1) Protein sequence of Nog1
2) Charge distribution
3) Disordered regions (GlobPlot) and low complexity region (CDD)
4) Disordered regions (Meta-Disorder)
5) Transmembrane topology (hmmtop)
6) Transmembrane topology (TMHMM)
7) As before, shown is the second result with a score of 544
8) Transmembrane topology (PHDhtm)
9) Transmembrane topology (BCL::Jufo9D, protein.jufo9d_tmh)
10) Protein binding sites (ISIS)
11) Beta-turns (NetTurnP)
12) Secondary structure (SOPMA)
13) Secondary structure (PSIPRED)
14) Secondary structure (Pred2ary via Medor)
15) Secondary structure (GOR4 via NPS@)
16) Secondary structure (PROFseq via PredictProtein)
17) Secondary structure (BCL::Jufo9D)
18) Secondary structure (Porter)
